# Nation-Wide Survey Assessing the Knowledge and Attitudes of Romanian Pharmacists Concerning Pharmacogenetics

**DOI:** 10.3389/fphar.2022.952562

**Published:** 2022-07-01

**Authors:** Cristina Pop, Anamaria Cristina, Irina Iaru, Stefan L. Popa, Cristina Mogoșan

**Affiliations:** ^1^ Department of Pharmacology, Physiology and Pathophysiology, Faculty of Pharmacy, Iuliu Hațieganu University of Medicine and Pharmacy, Cluj-Napoca, Romania; ^2^ 2nd Medical Department, Iuliu Hațieganu University of Medicine and Pharmacy, Cluj-Napoca, Romania

**Keywords:** pharmacy education, pharmacogenetics, pharmacogenomics, genetic variability evaluation, drug therapy optimization, drugs’ benefit-risk assessment

## Abstract

**Background:** Pharmacogenetics (PGx) is an important component of personalized medicine that has the potential to improve medicines’ effectiveness and safety. However, despite progress in technology and availability, PGx testing application into patient-care in Eastern Europe countries, has been slow.

**Objectives:** Our aim was to describe knowledge and attitudes of Romanian pharmacists concerning PGx, and identify potential factors limiting PGx implementation.

**Method:** An anonymous, web-based questionnaire was distributed to Romanian pharmacists registered in the National Pharmacists’ Association (NPA) *via* an official e-mail sent by NPA representatives.

**Results:** A total of 1,058 pharmacists completed the questionnaires, resulting in a response rate of 7.6%. Pharmacists were predominantly female (90.1%), younger than 49 years (87.5%) and mostly worked in community pharmacies (80.2%). Most pharmacists (64.8%) had a knowledge score between 30 and 49 points out of 60, and (75.4%) had attitude scores between 9 and 7 out of 10. Attitude and knowledge scores positively correlated.

**Conclusion:** Despite performing fairly well on general questions regarding PGx, Romanian pharmacists may lack in-depth knowledge, which can affect their readiness to discuss PGx information with patients or other healthcare professionals. High pricing was considered an important impediment in PGx implementation.

## Introduction

One of the most revolutionizing concepts included in personalized medicine is the use of patient genetic data to help make optimal decisions regarding drug therapy (Wang et al., 2020). Pharmacogenetics and pharmacogenomics are important scientific fields that study the influence of genetic factors over the efficacy and safety of medicines (Bishop et al., 2018; Elewa and Awaisu, 2019). The abbreviation PGx has been used to designate either pharmacogenetics or pharmacogenomics, which are often used interchangeably in the context of precision medicine when discussing the impact of genetic testing over drug therapy decision-making (Wang et al., 2020). Because pharmacogenetics is currently more approachable, due to the relatively decreased complexity in assessment of genetic variability and the drug-gene possible interactions, it is both the preferred clinical approach and the most used term (Barbarino et al., 2018). However, use of high-throughput analysis machines on a larger scale and in clinical settings, together with powerful decision-making software may soon enable healthcare providers to use pharmacogenomics in drug-related decision-making on a regular basis (Gammal et al., 2019). Genetic variations are at the base of interindividual variability in drug response, that may result in adverse drug reactions or lack of drug efficacy (Hansen et al., 2021). Adverse drug reactions (ADRs) may cause discomfort, lower the quality of life and alter medication adherence, or even worse, cause hospital admission, morbidity and mortality. All of these attract great healthcare resource use and high costs on the healthcare system. It is estimated that 20% of patients will suffer an ADR in their lifetime, and up to 30% of them may be the cause of hospital admission (Cacabelos et al., 2021). Drug response variability may increase both the incidence and the severity of ADRs. PGx may be responsible for about 20–95% of drug response variability and from all ADRs leading to hospital admissions, around 30% are caused by drugs with PGx implications (Cacabelos et al., 2021). On the other side, lack of drug therapy efficacy could also be the cause of complications, leading to an estimated 37–44% increase in hospital admission and even higher costs (Kitzmiller et al., 2011).

PGx is rapidly evolving and it is increasingly challenging for healthcare providers to keep up with the new information on PGx testing that impacts the prescription and clinical monitoring of drug therapy (Gammal et al., 2019). Currently, PGx information is included in the labels of almost 400 drugs by the US Food and Drug Administration and of over 150 drugs by the European Medicines Agency (Clinical guideline annovations, 2022). Also, among the most prescribed 50 drugs, 48% have PGx label information (Fuentes et al., 2018).

As point-of-care providers and drug experts, pharmacists are uniquely positioned in the healthcare system to appreciate the necessity of ordering a PGx test and to interpret the results for an optimal adjustment of patient drug therapy (Haidar et al., 2022). Therefore, pharmacists could play an important role in the integration of genotype-guided drug therapy into routine practice. However, in order to do this, pharmacists should be knowledgeable about PGx and be willing to communicate PGx information both to patients and to other healthcare partners (Tuteja et al., 2013).

Despite their many benefits to patients and the healthcare system, and despite the fact that many medical centers have started to offer direct-to-consumer PGx testing, their application into patient care in developing countries, including Eastern European countries, has been slow to implement (Gammal et al., 2019).

Important factors inhibiting a wider use of PGx testing could be the lack of knowledge and confidence concerning PGx, lack of access to testing, insufficient evidence and guideline implementation, data security concerns, technical difficulties, delay between sample collection and test result and last but not least, the cost of testing and data interpretation (Nagy et al., 2020; Wang et al., 2020).

Currently, there is limited research within the field of attitudes and knowledge regarding PGx in the context of primary care (Hansen et al., 2021). Thus, the objective of our study was to describe pharmacists in Romania, in terms of their knowledge and attitudes toward PGx. Even if evaluation of healthcare professionals’ knowledge and attitudes in other European countries has been undertaken (Pisanu et al., 2014; Just et al., 2017), to the best of our knowledge, this is the first study on a nation-wide scale to investigate pharmacists’ opinions and knowledge concerning PGx in an Eastern European country.

## Materials and Methods

### Study Design

We performed an anonymous, nation-wide cross-sectional survey between March and May 2017, using a web-based questionnaire. An e-mail invitation with a link to the online survey through the web-based survey tool, Google forms (CA, USA), was distributed to the Romanian pharmacists registered in each of the 41 Regional Pharmacists’ Associations in the Country (regional professional associations for pharmacists, present in each county and part of the Romanian National Pharmacists’ Association), to approximately 14.000 pharmacists (ref). Two reminder e-mails were sent 4 weeks apart. The invitation also presented the aims of the study and highlighted the fact that completion of the questionnaire is voluntary, anonymous and represents the agreement to participate in the study.

### The Survey Questionnaire

The questionnaire consisted of 24 questions. Questions regarding pharmacists’ demographic and practice characteristics (*n* = 7) and questions assessing practices and educational preferences regarding PGx (*n* = 2) were created by the authors. Questions focusing on attitude towards PGx (*n* = 5) were adapted from the study of *Tuteja et al.* (Tuteja et al., 2013) and questions concerning knowledge (*n* = 10) were partially created by the authors and partially adapted from the study of *Bannur et al.* (Bannur et al., 2014).

We used the questions assessing knowledge regarding PGx to calculate a knowledge score. Each question was assigned a maximum of 6 points (resulting in a total of 60 points).

Attitude-assessing questions were included in the attitude score calculation. Each answer would receive points, as follows: “Yes”—2 points; “Neutral”—1 point; “No”—0 points. A maximum of 10 points for attitude regarding PGx was possible.

In order to evaluate practices regarding PGx, pharmacists were asked if “PGx information had been requested in their practice” and for educational preferences, pharmacists were asked about their preferred way to receive further training in the PGx field: either “workshop”, “online course” or “conferences”.

Once the questions were created and translated into Romanian, we invited two of our expert colleagues and collaborators who understand the topic to go through the questionnaire, read it and fill all the questions. Our requirement was to carefully evaluate all the questions, to check for the confusing ones, the repetitive questions or the ones that could have multiple meanings. Also, to identify and verify the leading questions and less important ones, whether they effectively capture the topic of interest, and their construction. Once we received the remarks, we adjusted the questionnaire, eliminated approximately 4 questions and tested it online among our pharmacy residents (around 30 residents). After receiving their comments, which were very few, we finalized the questionnaire.

### Statistical Analysis

The collected data were analyzed using SPSS version 23.0 statistic software package. Descriptive statistics were generated as a whole and by type of pharmacy education: Final year Pharmacy student, Bachelor of Science in Pharmacy, Resident pharmacist, Specialist pharmacist, Primary pharmacist. The total knowledge and attitude scores were expressed as mean ± standard deviation (SD), and were analyzed by one-way ANOVA followed by Games-Howell post hoc test. Results were considered statistically significant if *p* value was 0.05 or less (*p ≤ 0.05*)*.*

## Results

### Characteristics of Study Participants

A total of 1,058 questionnaires were received, and because it is estimated that at the time of the survey distribution there were approximately 14.000 pharmacists registered in the RPA in Romania, this leads to a study response rate of 7.6%. Demographic information of the study participants is summarized in Table 1. Participants were either Bachelor of Science (BS) pharmacists (64.7%), Resident pharmacists (4.7%), Specialist pharmacists (12.6%) or Primary pharmacists (11.8%). Most pharmacists were female (90.1%) and younger than 49 years (87.5%). Additionally, besides their BS degree, 6.9% of the pharmacists had a Master’s degree and 4.5% a PhD. Some of the study participants were final year pharmacy students (6.2%) who were doing their pharmacy practice.

**TABLE 1:** Demographics of survey participants.

Characteristics	Total; n (%)	BS pharm; n (%)	Resident pharm; n (%)	Specialist pharm; n (%)	Primary pharm; n (%)	Other; n (%)
Gender						
Male	88 (8.3)	61 (9.3)	3 (6.0)	11 (8.3)	9 (7.2)	2 (3)
Female	953 (90.1)	591 (89.8)	47 (94.0)	121 (91.0)	114 (91.2)	64 (97)
Unknown	17 (1.1)	6 (0.9)	0 (0.0)	1 (0.8)	2 (1.6)	0 (0.0)
Age category (years)						
<30	381 (36.0)	249 (37.8)	44 (88.0)	22 (16.5)	4 (3.2)	60 (90.9)
30–39	278 (26.3)	222 (33.7)	5 (10.0)	29 (21.8)	10 (8.0)	2 (3.0)
40–49	267 (25.2)	163 (24.8)	1 (2.0)	52 (39.1)	43 (34.4)	4 (6.0)
50–59	67 (6.3)	17 (2.6)	0 (0.0)	21 (15.8)	26 (20.8)	0 (0.0)
>59	52 (4.9)	3 (0.5)	0 (0.0)	9 (6.8)	40 (32.0)	0 (0.0)
Unknown	13 (1.2)	4 (0.6)	0 (0.0)	0 (0.0)	2 (1.6)	0 (0.0)
Level of professional training						
Msc Pharm	73 (6.9)	54 (8.2)	3 (6.0)	6 (4.5)	10 (8.0)	0 (0.0)
PharmD	48 (4.5)	25 (3.8)	2 (4.0)	12 (9.0)	9 (7.2)	0 (0.0)
Professional experience (years)						
0–5	418 (39.5)	285 (43.3)	41 (82.0)	24 (18.1)	7 (5.6)	57 (83.4)
6–10	257 (24.3)	215 (32.7)	9 (18.0)	16 (12.0)	8 (6.4)	4 (6.1)
11–20	213 (20.1)	137 (20.8)	0 (0.0)	41 (30.8)	26 (20.8)	1 (1.5)
>20	156 (14.7)	21 (3.2)	0 (0.0)	52 (39.1)	82 (65.6)	0 (0.0)
Unknown	14 (1.3)	0 (0.0)	0 (0.0)	0 (0.0)	2 (1.6)	4 (6.1)
Primary work setting						
Independent pharmacy	472 (44.6)	300 (45.6)	12 (24.0)	68 (51.1)	67 (53.6)	18 (27.3)
Chain pharmacy	387 (35.6)	293 (45.5)	23 (46.0)	31 (23.3)	17 (13.6)	13 (19.7)
Hospital pharmacy	60 (5.7)	13 (2.0)	6 (12.0)	13 (9.8)	28 (22.4)	0 (0.0)
University	71 (6.7)	13 (2.0)	8 (16.0)	10 (7.5)	6 (4.8)	34 (51.5)
Pharmaceutical Company	51 (4.8)	31 (4.7)	2 (4.0)	8 (6.0)	4 (3.2)	4 (6.1)
Other	28 (2.6)	10 (1.5)	3 (6.0)	6 (4.5)	5 (4.0)	4 (6.1)
Unknown	22 (2.1)	11 (1.7)	1 (2.0)	1 (0.8)	1 (0.8)	1 (1.5)
Area of activity						
Rural	136 (12.9)	92 (14.0)	3 (6.0)	20 (15.0)	11 (8.8)	7 (10.6)
Urban, < 50.000 residents	262 (24.8)	179 (27.2)	5 (10.0)	22 (16.5)	35 (28.0)	16 (24.2)
Urban, >50.000 residents	645 (61.0)	387 (58.8)	42 (84.0)	90 (67.7)	77 (61.6)	39 (50.1)
Unknown	15 (1.4)	0 (0.0)	0 (0.0)	1 (0.8)	2 (1.6)	4 (6.1)
Geographical region						
West Romania (Banat)	87 (8.2)	55 (8.4)	2 (4.0)	13 (9.8)	11 (8.8)	4 (6.1)
South-East Romania (Dobrogea)	89 (8.4)	82 (12.5)	0 (0.0)	1 (0.8)	1 (0.8)	5 (7.6)
North-East Romania (Moldova)	233 (22.0)	150 (22.8)	1 (2.0)	29 (21.8)	39 (31.2)	8 (12.1)
South-Romania (Muntenia)	224 (21.2)	127 (19.3)	10 (20.0)	32 (24.1)	32 (25.6)	15 (22.7)
South-West Romania (Oltenia)	54 (5.1)	33 (5.0)	2 (4.0)	11 (8.3)	4 (3.2)	3 (4.5)
Center, North and North-West Romania (Transilvania)	362 (34.2)	209 (31.8)	35 (70.0)	47 (35.3)	38 (30.4)	31 (47.0)
Unknown	9 (0.9)	2 (0.3)	0 (0.0)	0 (0.0)	0 (0.0)	0 (0.0)

Not every participant answered every question.

BS, bachelor of science; PharmD, doctor of pharmacy; MSc Pharm, Master degree in Pharmacy; Pharm, Pharmacist.

Professional experience of pharmacists was mostly under 5 years (39.5%), followed by 6–10 years (24.3%) and 11–20 years (20.1%). The majority of pharmacists worked in community pharmacies (80.2%), followed by university (6.7%), hospital pharmacies (5.7%) and industry (4.8%) settings. Regional distribution showed that most of the respondents worked in urban areas (85.7%), rather than rural (12.9%). Additionally, we performed an analysis concerning the six main geographical regions of Romania and evaluated distribution of respondents as follows: West Romania (Banat) (8.2%), South-East Romania (Dobrogea) (8.4%), North-East Romania (Moldova) (22.%), South-Romania (Muntenia) (21.2%), South-West Romania (Oltenia) (5.1%) and Center, North and North-West Romania (Transylvania) (34.2%).

### Pharmacists’ Knowledge Concerning PGx

Table 2 shows participants’ answers to specific questions regarding the field of PGx. A vast majority of pharmacists were aware that PGx studies the influence of genetic variability over drug efficacy and safety (82%), that genetic variations can account for drugs’ efficacy (96.1%) and adverse reactions (88%). However, pharmacists did not know to such great extent that PGx influences pharmacokinetics and pharmacodynamics of drugs (37.6%), that direct-to-consumer PGx testing is already available in laboratories in Romania (22.4%) and that PGx information has already been included in the Summary of Product Characteristics (SmPCs) of some medicines (34.6%).

**TABLE 2:** Pharmacists’ knowledge of PGx.

Statement	Answered correctlyN (%)	Answered incorrectlyN (%)	UnknownN (%)
1. Pharmacogenetics studies the influence of genetic variability over drugs’ efficacy and/or safety	856 (82.0)	188 (18.0)	14 (1.3)
2. Pharmacogenetics can influence drugs’ pharmacokinetics, pharmacodynamics	392 (37.6)	653 (61.7)	14 (1.3)
3. Genetic variations can account for drugs’ efficacy	1,017 (96.1)	41 (3.9)	0 (0.0)
4. Pharmacogenetics testing is already available in laboratories in our country	237 (22.4)	821 (77.6)	0 (0.0)
5. Pharmacogenetics information is already available in the SmPCs of some drugs	366 (34.6)	692 (65.4)	0 (0.0)
6. Genetic variability can be a risk factor for adverse drug reactions	931 (88.0)	127 (12.0)	0 (0.0)
7. Clinical implementation of pharmacogenetics can increase drug therapy efficacy	893 (85.3)	154 (14.7)	11 (1.1)
8. Clinical implementation of pharmacogenetics can decrease the number of adverse reactions to drugs	851 (81.5)	193 (18.5)	14 (1.3)
9. Pharmacogenetics is an important component of personalized therapy	904 (86.4)	142 (13.6)	12 (1.2)
10. Genetic variability cand influence the efficacy and safety of the following drugs			
Clopidogrel	517 (48.9)	541 (51.1)	0 (0.0)
Codeine	285 (29.9)	773 (73.1)	0 (0.0)
Combined oral hormonal contraceptives	763 (72.1)	295 (27.9)	0 (0.0)
Carbamazepine	508 (48.0)	550 (52.0)	0 (0.0)

SmPCs, Summary of Product Characteristics.

Most pharmacists were aware that clinical implementation of PGx can increase drug therapy efficacy (85.3%) and can decrease the number of adverse reactions (81.5%). Also, most pharmacists (86.4%) were aware that PGx is an important component of personalized therapy. When asked more specific questions regarding drugs, most pharmacists (72.1%) knew that combined oral hormonal contraceptives safety can be influenced by genetic variability, but were mostly unaware that clopidogrel (48.9%), codeine (29.9%) and carbamazepine (48%) efficacy or safety can be influenced by genetic variability.

Regarding pharmacists’ knowledge concerning PGx, Table 3 presents the classification of calculated scores, categorized by demographic characteristics. A total of 224 (21.2%) participants had an above-average score of ≥50 out of 60 points for the assessment of knowledge on PGx, while the majority (686 participants, 64.8%) had a score between 30 and 49 points and 138 (13%) participants had a score below 29 points.

**TABLE 3:** Knowledge and attitude scores association with respondents characteristics (*n* = 1,058)^†^.

Characteristics	Total; n (%)	Total Knowledge Score	Total Attitude Score
		Mean ± SD	Mean ± SD
Gender			
Male	88 (8.3)	43.3 ± 9.4	7.42 ± 1.5
Female	953 (90.1)	42.8 ± 8.7	7.42 ± 1.4
Age category (years)			
<30	381 (36.0)	43.3 ± 9.0***	7.4 ± 1.5
30–39	278 (26.3)	41.9 ± 8.8***	7.5 ± 1.5
40–49	267 (25.2)	41.4 ± 7.3***	7.4 ± 1.3
50–59	67 (6.3)	43.1 ± 9.7***	7.2 ± 1.5
>59	52 (4.9)	42.0 ± 8.3**	7.6 ± 1.3
Level of professional training			
Student	66 (6.2)	43.0 ± 8.0**	7.1 ± 1.6
BS Pharm	658 (62.2)	42.9 ± 7.3**	7.4 ± 1.4
Resident Pharm	50 (4.7)	46.0 ± 7.9***	7.9 ± 1.1^##^
Specialist Pharm	133 (12.6)	44.2 ± 8.8***	7.6 ± 1.5
Primary Pharm	125 (11.8)	43.6 ± 8.0***	7.6 ± 1.5
MSc Pharm	73 (6.9)	44.2 ± 9.5***	7.5 ± 1.3
PharmD	48 (4.5)	47.6 ± 8.6***	8.1 ± 1.1^#^
Professional experience (years)			
0–5	418 (39.5)	43.1 ± 8.9***	7.4 ± 1.5
6–10	257 (24.3)	41.3 ± 8.4***	7.4 ± 1.2
11–20	213 (20.1)	42.5 ± 8.9***	7.5 ± 1.5
>20	156 (14.7)	42.5 ± 8.6***	7.4 ± 1.4
Primary work setting			
Community pharmacy	826 (78.1)	40.1 ± 10.9	7.4 ± 1.4
Hospital pharmacy	60 (5.7)	44.6 ± 7.2***	7.5 ± 1.3
University	71 (6.7)	45.7 ± 8.4***	7.6 ± 1.4
Pharmaceutical Company	51 (4.8)	45.8 ± 7.8***	7.9 ± 1.3^¥^
Other	28 (2.6)	45.3 ± 7.7**	7.4 ± 1.6
Area of activity			
Rural	136 (12.9)	41.3 ± 10.1	7.5 ± 1.6
Urban, < 50.000 residents	262 (24.8)	42.1 ± 8.7	7.5 ± 1.3
Urban, >50.000 residents	645 (61.0)	40.8 ± 11.3	7.4 ± 1.4
Geographical region			
West Romania (Banat)	87 (8.2)	41.6 ± 8.1***	7.1 ± 1.3
South-East Romania (Dobrogea)	89 (8.4)	40.6 ± 8.4***	7.2 ± 0.7
North-East Romania (Moldova)	233 (22.0)	41.9 ± 9.4***	7.6 ± 1.3^‡‡^
South-Romania (Muntenia)	224 (21.2)	43.4 ± 8.3***	7.6 ± 1.4^‡^
South-West Romania (Oltenia)	54 (5.1)	40.0 ± 12.3***	6.9 ± 2.3
Center, North and North-West Romania (Transilvania)	362 (34.2)	43.7 ± 8.3***	7.4 ± 1.4

*p < 0.05, **p < 0.025, ***p < 0.01; #*p* < 0.05, PharmD *vs*. MSc Pharm; ##*p* < 0.025, Resident Pharm *vs*. BS Pharm; ¥*p* < 0.05, Pharmaceutical company *vs*. Community pharmacy; ‡*p* < 0.05 Muntenia *vs*. Banat and Muntenia *vs*. Dobrogea; ‡‡*p* < 0.025 Moldova *vs*. Dobrogea; †Not every participant answered every question; BS, Bachelor of science; PharmD, Doctor of Pharmacy; MSc Pharm, Master degree in Pharmacy; Pharm, Pharmacist.

There was no difference in knowledge scores between genders. The highest knowledge scores were obtained by pharmacists that were younger (aged <30 years), had under 5 years of work experience, were still in training as resident pharmacists and those that had a PhD title. The knowledge differences between participants, concerning the level of professional training, were not significant for residents (46 ± 7.9), specialists (44.2 ± 8.8) and primary pharmacists (43.6 ± 8), when compared to each other, but they were significantly higher when compared to BS pharmacists (39.7 ± 11.2). Pharmacists with a PharmD degree had the highest scores for knowledge (47.6 ± 8.6), compared to all the other groups. All professionals that worked in hospital pharmacies, university and pharmaceutical companies presented a statistically significant (*p* < 0.05) higher knowledge score (44.6 ± 7.2) (45.7 ± 8.4) and (45.8 ± 7.768), respectively, compared to those from community pharmacies (40.1 ± 10.9). Rural or urban setting of the pharmacy did not influence knowledge scores. Concerning geographical regions, respondents in Muntenia (South Romania) and Transylvania (Center, North and North-West Romania) presented significantly higher scores compared to respondents in other regions, while the lowest scores were observed for respondents working in Oltenia (South-West) and Dobrogea (South-East).

### Pharmacists’ Attitude and Practices Concerning PGx

The results shown in Table 4, highlight the fact that only 14.7% of pharmacists considered themselves ready to discuss PGx information with other medical professionals or patients and only 22.1% of pharmacists considered PGx information included in SmPCs as easily accessible to them. More than half of the participants (55.1%) agreed that high pricing could be an impediment in accessing PGx testing, while 39.7% stated not having sufficient knowledge regarding PGx testing pricing.

**TABLE 4:** Pharmacists’ attitude toward PGx.

Question	Yes N (%)	NeutralN (%)	No N (%)	UnknownN (%)
1. Do you feel ready to discuss with other medical professionals about a patient’s therapy in the context of PGx information?	155 (14.7)	489 (46.2)	401 (37.9)	—
2. Do you feel that PGx information currently available in the SmPCs of some medicines is easily accessible to you?	234 (22.1)	496 (46.9)	312 (29.5)	16 (1.5)
3. Do you believe that PGx information is useful for your profession?	975 (92.2)	62 (5.9)	9 (0.9)	—
4. Do you believe that including pharmacogenetics in the curriculum of the Faculty of Pharmacy is important?	954 (90.2)	76 (7.2)	13 (1.2)	15 (1.4)
5. Are you willing to learn more about pharmacogenetics?	1,008 (95.3)	31 (2.9)	4 (0.4)	15 (0.7)

On the other hand, most pharmacists acknowledged the usefulness of PGx for their profession (92.2%), considered it is important to study PGx during bachelor studies (90.2%) and expressed their interest to learn more about PGx (95.3%).

Some participants (5.5%) obtained a maximum attitude score (10/10 points), while the majority (75.4%) had scores between 9 and 7, and 19.1% had scores below 6 points.

Table 3 presents the attitude scores categorized by demographic variables. Gender, age and professional experience did not influence attitude scores. A statistically significant (*p* < 0.05) higher attitude score was observed in pharmacists working in pharmaceutical companies (7.9 ± 1.3), compared to professionals from community pharmacies (7.4 ± 1.4). Also, a statistically significant higher attitude score for pharmacists in residency training compared to BS pharmacists was observed (7.9 ± 1.1 versus 7.4 ± 1.4, *p* < 0.05). Pharmacists with PharmD degree presented with the highest scores for attitude (8.1 ± 1.1).

Rural or urban setting of the workplace did not influence attitude scores. There were no statistical differences concerning attitudes towards PGx in different geographical regions of Romania. However, pharmacists in Moldova and Muntenia presented the highest attitude scores (7.6 ± 1.3, 7.6 ± 1.4, respectively), while pharmacists in Dobrogea and Banat presented the lowest scores (7.2 ± 0.7, 7.1 ± 1.3, respectively).

The preferred means of improving knowledge concerning PGx were mainly through online courses (65.7%), followed by pharmaceutical conferences (56.4%) and workshops (35%).

Regarding their practice, one additional question to pharmacists was if they had ever been asked information regarding PGx by their patients, for which only 16.5% of pharmacists answered affirmatively.

## Discussion

Globally, PGx has constantly evolved, with efforts being made in research, regulatory affairs and commerce for a continuously increased implementation and translation of PGx information into practice. As the effect of gene variability on drug efficacy and safety has been elucidated for many drugs, various organizations have created evidence-based practice guidelines that are being constantly updated and curated by the pharmacist-led organization known as the Pharmacogenomics Knowledge Base (PharmGKB) (Gong et al., 2021). However, the information presented by platforms such as PharmGKB and even information included in the SMPCs of medicines can be difficult to understand.

With the evolution of PGx, pharmacists’ role in implementing PGx will develop. The importance of pharmacists’ involvement in PGx is currently being highlighted by a diversity of initiatives. In a statement draft on the pharmacist’s role in clinical PGX, the American Society of Health-System Pharmacists (ASHP) defined the pharmacists’ possible responsibilities concerning PGx services (Haidar et al., 2022). Moreover, the American Pharmacists Association has also acknowledged the pharmacists’ capacity to incorporate PGx into drug therapy management, while the American College of Clinical Pharmacy and the American Association of Colleges of Pharmacy host PGx practitioner and educator groups (Owusu-Obeng et al., 2014).

Nonetheless, the International Pharmaceutical Federation (FIP) has recently organized a special interest group focusing on personalized and precision medicine. The objectives of this group are to foster scientific exchange in the area of precision medicine among pharmacists, to keep pharmacists up-to-date with the latest research in this area, to help pharmacists optimize treatment selection based on precision medicine approaches and to highlight the usefulness of precision medicine by presenting examples of successful practical implementation (International Pharmaceutical Federation, 2022).

Although direct-to-consumer PGx testing has been available for some time in Romania, the prescription and implementation of PGx services have been slow. By evaluating the knowledge and attitude of pharmacists in Romania, concerning PGx we could extrapolate the reasons behind this slow PGx implementation and try to find future directions for improvement.

As our aim was to present a national view on knowledge and attitude towards PGx, we believed that an online questionnaire would be the most time- and cost-effective means. Although for a nation-wide questionnaire, the response rate of about 7.6% was acceptable, the obtained response rate was due primarily to the means of questionnaire invitation (*via* e-mail). In a study performed in the Netherlands, using formerly pregnant women as subjects, the authors considered that even though the web-based method of survey administration is a fast and easy way to access participants, it also is the most important cause of low response rate due to the less personal mode of contact, technical difficulties and limited availability of time (International Pharmaceutical Federation, 2022).

Concerning demographic characteristics of participants, mostly were female, which is influenced by two important aspects: 1) in Romania the vast majority of pharmacists are female (Sandulovici et al., 2018) and 2) females seem to be more receptive towards surveys (Smith and San, 2008).

Moreover, the vast majority of pharmacists were younger than 49 years old, with little (under 5 years) professional experience, suggesting both a higher interest in this modern field and a higher familiarity with the technical aspects concerning an online questionnaire. Also, studies show that young people are more likely to participate in surveys (Smith and San, 2008).

Additionally, most pharmacists worked in community pharmacies, from urban areas. It was expected to receive the most responses from community pharmacists as in Romania, most pharmacists work in community pharmacies, whereas hospital and clinical pharmacists represent a very small proportion of pharmacists (Sandulovici et al., 2018). Most responses were received from pharmacists registered in Moldova, Muntenia and Transylvania, which are the main three regions of Romania, with the highest population density and also highest proportion of registered pharmacists (Sandulovici et al., 2018).

Concerning pharmacists’ knowledge regarding PGx, we found that despite the majority of pharmacists having had no special training in PGx, they scored relatively high on the knowledge survey. Most pharmacists knew that PGx studies the influence of genetic variability over efficacy and safety of drugs. Similar results were observed in other studies performed in high-income countries (Haga et al., 2021), but in middle- and low-income countries pharmacists’ knowledge concerning PGx was lower, with fewer professionals being familiar with this field (Nagy et al., 2020; Albitar and Alchamat, 2021; Mufwambi et al., 2021). However, when asked more specific questions, Romanian pharmacists displayed lower knowledge, not being aware of the mechanisms behind drug-response variability or regarding the influence of genetic variability over the efficacy and safety of drugs such as clopidogrel, codeine or carbamazepine. Additionally, most pharmacists were not aware that PGx testing is commercially available in Romania and that PGx information was already included in the SmPCs of many drugs. Similar to other studies, highest scores were obtained by younger pharmacists, with lower work experience, and presenting additional training (residency or PhD), besides BS (Hansen et al., 2021). Usually, younger pharmacists and those still in training have several advantages such as recent contact with information concerning PGx during university and post-university studies, a more open attitude towards new and innovative fields of healthcare and also towards technology. The curricula in Pharmacy Faculties in Romania have not included, so far, specific mandatory courses on PGx. However, many disciplines, such as Pharmacology, Molecular biology and others have adapted their courses to include PGx information. Future development and implementation of specific courses regarding PGx could increase pharmacists’ knowledge in this field. However, in a study evaluating students’ attitudes and perceptions concerning PGx education in a developed country, only 40% felt that it had been a relevant part of their training, 46% stating that they had received only 1–3 lectures on PGx and the majority did not feel ready to use the information they accumulated in practice (Coriolan et al., 2019). One other category of pharmacists that presented with higher knowledge scores are pharmacists that have a PhD title. However, this would be expected as they usually work in universities or research institutes and are constantly in contact with the new aspects of pharmaceutical sciences.

Interestingly, pharmacists working in rural areas did not present lower knowledge scores compared to pharmacists in urban areas. This could be explained by the fact that most pharmacists in Romania that work in rural areas, actually commute from urban areas and most rural-based pharmacies are chain pharmacies that constantly change the pharmacists that commute to rural areas.

Concerning geographical regions, respondents in Muntenia and Transylvania presented with the highest knowledge scores probably due to the proximity of three of the biggest Faculties of Pharmacy (Bucharest, Cluj-Napoca and Târgu Mureș), as most pharmacists tend to start working in pharmacies in or close to the university center where they had studied (Sandulovici et al., 2018). We hypothesized that by working in the proximity of the academic center, pharmacists are better informed and more motivated to invest in their professional development and to be actively involved in research studies, to ensure that they provide optimal patient care, given that the three academic centers are also the most important medical centers in the country. For pharmacists working in university cities, attending continuous education courses and being up-to-date with the newest science is usually easier. However, the COVID-19 pandemic has brought a surge in offers for online courses as a response to social distancing measures. Thus, we could expect that the easier access to courses would improve knowledge in many new fields of pharmaceutical sciences in the future.

Despite not performing low on knowledge scores, pharmacists did not consider themselves ready to discuss PGx information neither with other health professionals, nor with patients. The low pharmacists’ confidence concerning their ability to deliver PGx testing, communicate with patients, and work with prescribers to appropriately act upon PGx results was also highlighted by the work of *Hansen et al.* who analyzed 15 studies and concluded that healthcare professionals feel unprepared to apply PGx to their clinical decision-making mainly because of low knowledge (Hansen et al., 2021). It is also possible that pharmacists also see this activity as a supplemental task in an already very demanding profession. This aspect has been evaluated in other studies showing that busy community pharmacies may not be able to provide in-depth PGx counseling due to the additional time requirements (Haga et al., 2021). However, this activity should be taken into consideration by pharmacies; direct-to-consumer PGx testing may also significantly increase the role of pharmacists as patients may consult with them to better understand their test results and how it impacts their current medication (Haga et al., 2021). Pharmacists can play a substantial role in identifying eligible patients, providing patient counseling before and after testing, sample collection and shipment, results interpretation, and communication with prescribers (Haga et al., 2021). Nevertheless, in our study, a fairly small number of respondents (16.6%) stated that they have been asked questions regarding PGx by their patients. This could represent one cause for the limited knowledge of pharmacists in the field of PGx. Not existing the demand for certain services may inhibit the development of professionals’ knowledge in the specific field. Thus, future training courses and information campaigns in PGx could also target other healthcare professionals such as doctors, who represent the main prescribers of PGx testing, thus assuring a higher demand of these tests and all the associated services, where pharmacists could also contribute.

More than half of the pharmacists stated that one impediment to the wider availability of PGx testing could be the high prices and the lack of reimbursement. Not only in lower-income countries, but also in higher-income countries testing pricing was found to be among the most important barrier in PGx testing implementation (Tuteja et al., 2013). However, in a recent analysis of studies that evaluated cost-effectiveness of PGx-guided treatment, more than 57% of studies concluded in favor of PGx testing. Additionally, 75% of studies found PGx-guided treatment as cost-effective and cost-saving (Verbelen et al., 2017). Nevertheless, as the cost of PGx tests is expected to decrease in the future, we can speculate that more patients would benefit from genetic testing and may require help interpreting and applying the results (Gammal et al., 2019). In this respect, our study shows that only a small proportion (less than a quarter) of the pharmacists that completed the survey considered PGx information included in SmPCs as easily accessible to them, thus being in great need of guidance and education in the field. Nevertheless, the great majority of responders acknowledged the usefulness of PGx for their profession and the importance of studying PGx both at university and as part of their continuing pharmaceutical education. Similar to our study, the work of Muzoriana *et al.* showed that most participants acknowledged the relevance of the field to their professional practice (74%) and confirmed the importance of teaching PGx in pharmacy schools (85%) (Muzoriana et al., 2017). However, another study showed that only 44% of the respondents (community pharmacists) considered pharmacogenetics relevant to their practice setting, mostly due to their busy schedule and the low prescribing of direct-to-consumer tests (Tuteja et al., 2013).

In line with other international studies, attitude scores of pharmacists regarding PGx were fairly high. As expected, a higher attitude score correlated with a higher knowledge score. However, there is the possibility of bias, as pharmacists with optimal knowledge and attitude were more willing to take the survey in the first place. In contrast to the influence that the professional experience seems to have on PGx specific knowledge, for attitude it is not an influencing factor. Similar to knowledge, work place setting (rural *vs*. urban) did not influence attitudes concerning PGx.

Similar to other studies, most of the pharmacists (96.3%) presented their interest in PGx education mainly through online courses, followed by pharmaceutical conferences or workshops (Bannur et al., 2014; Berenbrok et al., 2019). The study of Schwartz et al. showed that 97% of the hospital pharmacists would be interested in PGx-related continuing education and the preferred method was online training (85%), followed by training at a national annual conference (39%) (Schwartz and Issa, 2017).

As the role of pharmacists in counseling patients regarding drug therapy will evolve by incorporating PGx, pharmacists will need to be better prepared to understand and use PGx information for the benefit of the patient. While most Romanian pharmacists have a positive attitude towards PGx, they currently do not feel prepared to discuss PGx information with both patients and other healthcare providers. Our study highlighted the need for advanced and complex/in-depth education regarding PGx among pharmacists. While classic types of lectures have their benefits, they also have disadvantages, thus online and interactive courses are the most interesting for the majority of pharmacists. Also, the inclusion of courses focused on PGx in the curricula of Romanian Pharmacy Schools would benefit pharmacists and would dramatically improve knowledge. Last but not least, considering the cost and availability of PGx testing, solutions could be found by offering discounts or co-payment from private funds in order to help patients that would benefit from PGx testing get the appropriate care.

## Data Availability

The raw data supporting the conclusions of this article will be made available by the authors, without undue reservation.
